# Supplementary material for: Synergetic Effect of Potassium Oxysalts on Combustion and Ignition of Al/CuO Composites
Source: Nanomaterials (Basel). 2021 Dec 12;11(12):3366. doi: 10.3390/nano11123366 (PMC8705615; doi:10.3390/nano11123366)
Supplement: Supplementary file 1 [file nanomaterials-11-03366-s001.zip › nanomaterials-1475630-supplementary.pdf]

nanomaterials

Supplementary Materials

Synergetic Effect of Potassium Oxysalts on Combustion and Ignition of Al/CuO Composites

Xiaohang Ma, Wanjun Zhao *, Wei Le, Jianxin Li, Pengwan Chen and Qingjie Jiao

State Key Laboratory of Explosion Science and Technology, Beijing Institute of Technology, Beijing 10081, China; mmxhang@163.com (X.M.); 3120190196@bit.edu.cn (W.L.); Ljx19950204@126.com (J.L.); pwchen@bit.edu.cn (P.C.); jqj@bit.edu.cn (Q.J.)

* Correspondence: wanjunzhaowj@bit.edu.cn

The details of the heat release measurement through the diagrammatic sketch of the calorimeter test device, which is shown as following:

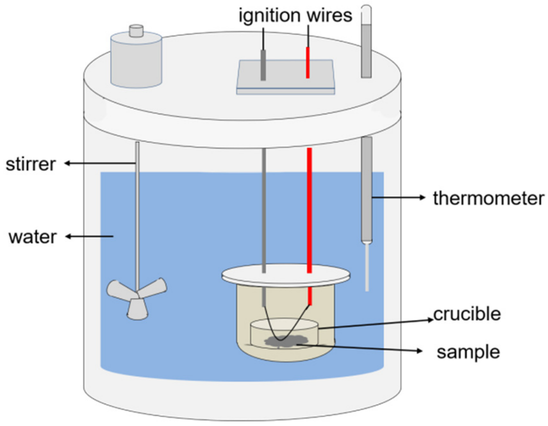

Figure S1. Diagrammatic sketch of the calorimeter test device.

The theoretical heat release of the composites is calculated under the assumption that the fuel and the oxidant completely react, but the composites couldn't completely react during the experiment. The combustion products of several composites were characterized by XRD. As shown in Figure S2, there are a small amount of Cu_2O and Al in the combustion products of Al/CuO, which means that the fuel and oxidant in the MIC can not react completely. Similarly, a small amount of residual Al in the product of Al/$KClO_4$ is not completely reacted, so the experimental heat release (8025 J g^{-1}) of Al/$KClO_4$ is less than the theoretical heat release (10,659 J g^{-1}).

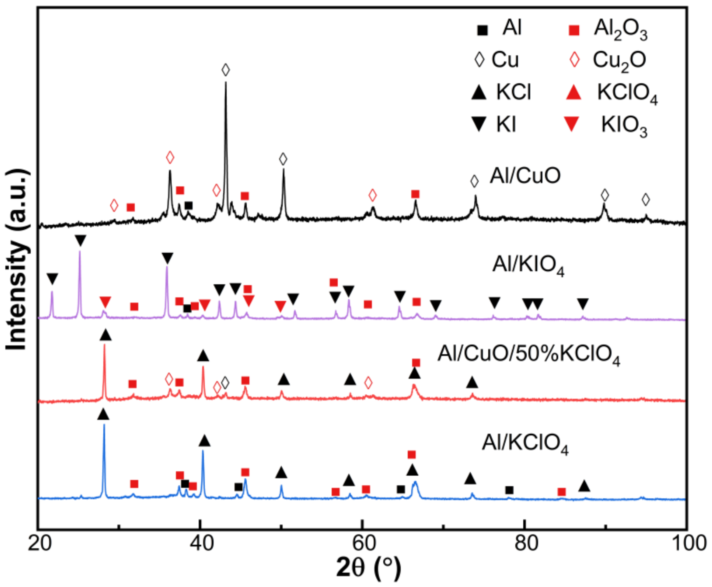

Figure S2. XRD characterization of the combustion products.
